# Cool Sex? Hibernation and Reproduction Overlap in the Echidna

**DOI:** 10.1371/journal.pone.0006070

**Published:** 2009-06-29

**Authors:** Gemma Morrow, Stewart C. Nicol

**Affiliations:** School of Zoology, University of Tasmania, Hobart, Tasmania, Australia; University of Hull, United Kingdom

## Abstract

During hibernation there is a slowing of all metabolic processes, and thus it is normally considered to be incompatible with reproduction. In Tasmania the egg-laying mammal, the echidna (*Tachyglossus aculeatus*) hibernates for several months before mating in mid-winter, and in previous studies we observed males with females that were still hibernating. We monitored the reproductive activity of radio-tracked echidnas by swabbing the reproductive tract for sperm while external temperature loggers provided information on the timing of hibernation. Additional information was provided by camera traps and ultrasound imaging. More than a third of the females found in mating groups were torpid, and the majority of these had mated. Some females re-entered deep torpor for extended periods after mating. Ultrasound examination showed a developing egg in the uterus of a female that had repeatedly re-entered torpor. The presence of fresh sperm in cloacal swabs taken from this female on three occasions after her presumed date of fertilization indicated she mated several times after being fertilized. The mating of males with torpid females is the result of extreme competition between promiscuous males, while re-entry into hibernation by pregnant females could improve the possibility of mating with a better quality male.

## Introduction

Hibernation has been documented in species from a wide range of mammalian orders [1], and although originally thought to be an adaptation to the cold, hibernation is now considered to be an energy conserving strategy which different species employ in a range of ecological circumstances [2]. Hibernation is characterized by a reduction in body temperature (T_b_) which typically falls to within 1°C of ambient, and a very substantial, but size dependent, reduction in metabolic rate [1]. Because metabolic processes are slowed during hibernation it is generally considered to be incompatible with reproduction: hibernation prevents spermatogenesis in males [3], slows fetal development, delays parturition [4] and inhibits lactation [5].

Among Australian mammals many dasyurid marsupials enter daily torpor during pregnancy [6], but bats are the only mammalian group in which reproduction and deep, extended torpor (i.e. hibernation) are known to overlap. In temperate zone bats, which show an extensive period of winter torpor, the reproductive cycle is interrupted by hibernation [7]. A number of strategies, including sperm storage and delayed ovulation, allow gestation to be initiated on arousal from hibernation in spring although gametogenesis occurs in summer [7], [8]. However, the only species known to enter deep, prolonged torpor while pregnant is the North American hoary bat (*Lasiurus cinereus*) - in extreme spring weather conditions pregnant females showed bouts of deep torpor lasting up to 5.6 days [9].

The short-beaked echidna *Tachyglossus aculeatus* is distributed throughout southern and eastern New Guinea, mainland Australia, Tasmania, Kangaroo Island, and smaller offshore islands. It is the most common of the egg-lying mammals and is in fact the most widespread native Australian mammal [10]. Throughout their range echidnas show some degree of seasonal inactivity. In Tasmania (subspecies *T. a. setosus*) reproductively active males hibernate from mid February to mid June, while reproductively active females hibernate from early March until mid July [11], [12]. By contrast reproductively active adults of the Kangaroo Island subspecies (*T. a. multiaculeatus*) show reduced activity and only intermittent bouts of torpor between April and June [13]. Courtship behaviour also appears to differ between the two areas [14]. Kangaroo Island echidnas have been described as forming mating “trains” of up to 11 individuals with a period of competition between males and courtship lasting between 14–44 days [15]. After mating there is a gestation period of 22–24 days, after which the female normally lays a single egg [12], [15].

Since 1996 we have been studying a population of echidnas in the Tasmanian southern midlands, and on several occasions during the course of this study we found males with females which were torpid, or which subsequently re-entered hibernation. In order to examine more closely the relationship between hibernation and reproduction in Tasmanian echidnas we conducted a detailed investigation of radio-tracked echidnas during the 2007 and 2008 mating seasons.

## Results and Discussion

Over the two mating seasons we found 26 mating groups. The most common number of males in a group was one (15 mating groups), but on three occasions in 2008 females were found with four males. In ten of the mating groups the female was torpid and reacted slowly to stimuli; body temperatures of these torpid females ranged from 10 to 29°C. All males in mating groups were active and had normal (euthermic) body temperatures (ca 32°C [16]). Of five torpid females checked, four had sperm in their reproductive tract. Four of the females found torpid in mating groups were radio tracked and were observed to re-enter hibernation; three of these were later found in mating groups while euthermic and had fresh sperm recovered from their tracts.

**Figure 1:**
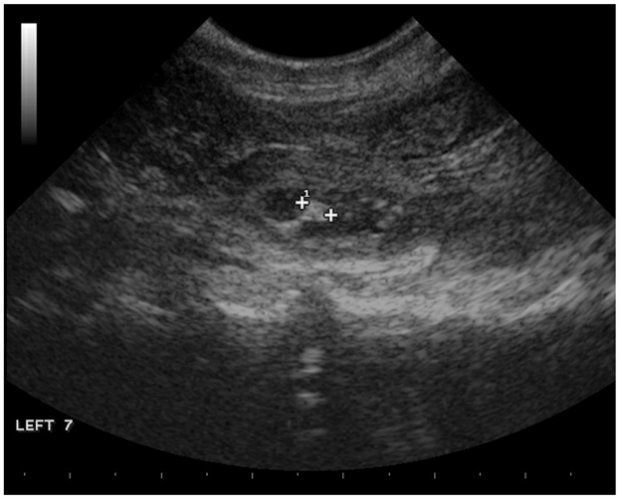
Ultrasound image showing an egg in the uterus of echidna 5D5E on July 23 2008. Fertilization probably occurred on July 9, but she had fresh sperm in her reproductive tract and was also torpid. Distance between the two markers showing the structure within the egg is 0.35 cm.

One female (echidna 5D5E) was studied intensively in 2008, using a combination of field observations (including cloacal swabbing), camera traps, an external temperature logger and ultrasound. This showed that while hibernating she was visited by a male at least twice without mating occurring. In one of these events (July 6) a camera trap showed that a male was with her in her hibernaculum for 13 hours, while the temperature logger showed that she did not rewarm significantly. She subsequently mated five times between July 11 and 28 before entering a nursery burrow on August 7. During the period of mating she repeatedly re-entered torpor with a minimum T_b_ of about 10°C. Maximum length of these torpor bouts was only about 12 hours, but she was being frequently disturbed by us. On July 15 female 5D5E was with male 5036, had fresh sperm in her tract, and had a temperature of less than 20°C. (A fault in the temperature probe prevented us from measuring her T_b_ more accurately). An ultrasound scan showed an egg in her uterus (Figure 1). A second scan 5 days later confirmed the presence of the egg, and showed fresh sperm again. Nine days before entering the nursery burrow she was with the same male, had fresh sperm in her tract, but was torpid with a temperature of 26.6°C.

**Figure 2:**
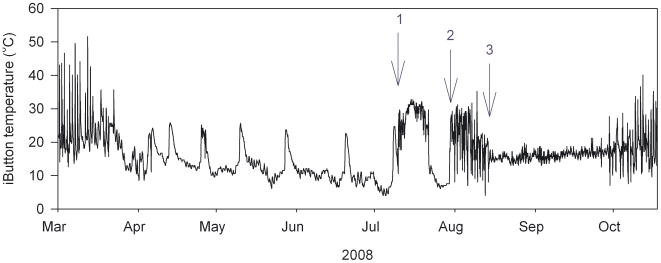
External temperature logger record from female echidna 0118. She entered hibernation in March, and her final arousal was on July 27 (arrow 2), when temperature variability increased. The subsequent reduction in variability (arrow 3) is associated with entry into the nursery burrow and egg-laying. Periodic arousals can be seen between April and July. In mid-July she shows an extended arousal (July 10–21), and we presume fertilization occurred at the time indicated by arrow 1.

**Figure 3:**
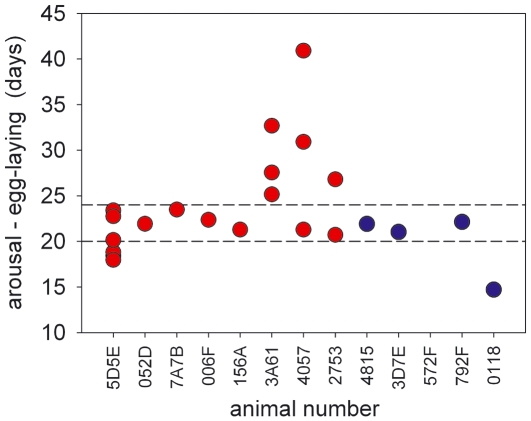
Time between final arousal from hibernation and egg-laying for 23 reproductive events from 13 echidnas, as estimated from internal (red circles), and external (blue circles) temperature loggers. The reported gestation period for echidnas is 20–24 days [15]. Although three animals (3A61, 4057, 2753) were active for periods of up to 3 weeks before becoming pregnant, the majority of points lie between 20 and 24 days after the end of hibernation. In these cases the females must have become pregnant nearly immediately after the final arousal from hibernation, or were already pregnant. Echidna 0118 must have become pregnant during the previous euthermic period (see Figure 2), as was probably also the case for three of 5D5E's pregnancies.

We have shown previously that internal body temperature loggers allow accurate timing of reproductive events [17], and as seen in Figure 2, this information can also be obtained from external temperature loggers. Figure 3 shows the time between final arousal from hibernation and egg-laying, as determined from these internal and external logger records for 21 reproductive events from 13 females, during this and previous studies. (Data from female 5D5E for 2008 have not been included as she was so frequently disturbed). The majority of eggs are laid between 20 and 24 days after the final arousal from hibernation. As the gestation period reported for echidnas is also 20–24 days [12], [15], this would indicate that most females become pregnant immediately after arousal from hibernation, or are already pregnant at their final arousal, as was the case for female 5D5E in 2008. Figure 3 shows that in some cases the time from final arousal to egg-laying is so short (e.g. female 0118) that there must have been considerable development of the egg before the final arousal. As seen in Figure 2, this female appears to have become pregnant during an extended euthermic period, and was probably euthermic for 8–9 days before re-entering hibernation for eight days, with T_b_ falling to about 7°C.

This is only the second account of a mammal entering deep torpor, or hibernation, when pregnant. In hoary bats it has been suggested that hibernation is used during harsh weather to delay parturition and thus lactation, which is more energetically expensive than pregnancy [9]. While this will be a significant consideration in hoary bats where the total litter mass is about 30% of maternal mass [18], the single newly hatched echidna young at about 0.5 g [19] will be less than 0.02% of maternal mass. In a previous study we found no measureable increase in field metabolic rates of lactating females with young aged 45–65 days [20], and thus the newly hatched baby will be an insignificant energy drain, at least initially. Furthermore, during the 10–11 days of egg incubation and first 30 days of lactation, Tasmanian echidnas are in a closed nursery burrow [12], and protected from the weather. Thus it seems unlikely that pregnant female echidnas enter deep torpor to postpone the energetic costs of lactation.

In our study area reproductively active males finished hibernation between May 10 and August 5 (*n* = 7), while the final arousal from hibernation of reproductively active females was between June 7 and September 3 (*n* = 23) [12]. As males are ready to mate about 30 days after the end of hibernation [12], many will be seeking matings while some females are still hibernating. Male mating activity lasts for about 60 days, and although we could not continually observe the animals, males seemed typically to stay with a female for up to seven days, with some males leaving and joining new groups, while others stayed in close proximity to the female for longer periods. Males were observed with up to four females during a breeding season, while females mated more than once, often with more than one male. Outside the mating season, echidnas are solitary and home ranges of males are typically twice that of females (Nicol SC, Vanpé C, Sprent JA, Morrow G, Andersen NA, unpublished observations). The mating trains noted on Kangaroo Island are also clearly a manifestation of intense competition between males, and, as the Tasmanian females are clearly promiscuous, the observation that female Kangaroo Island echidnas only mate once [15] is likely to be incorrect. Thus the echidna mating system appears to be characterised by roving promiscuous males [21] which guard promiscuous females before and after mating.

When females are polyandrous or promiscuous there is selection for male traits favoured by cryptic male choice, or traits that increase competitiveness during sperm competition [22]. Male traits that potentially increase fertilization success include genital morphology, sperm size and morphology, and copulatory and post-copulatory behaviour [22]. The male echidna has an elaborate penis which has a quadripartite anemone-like appearance [23], and ejaculates its sperm in bundles [24]. Sperm bundles are very likely to be an adaptation for sperm competition as spermatozoa in larger bundles show greater progressive motility than single spermatozoa or smaller sperm bundles [25]. Another trait that should increase competitiveness during sperm competition is large testes, and monotremes have larger testes relative to body size than marsupials, primates or avian species [26].

We suggest that there is extreme competition between echidna males, which in the Tasmanian sub-species leads to males mating with torpid females. A male finding a hibernating female, and repeatedly mating with her, and then guarding her, would have a high probability of successful paternity. Our observations raise the possibility that the echidna is an induced ovulator - in induced ovulators copulation initiates ovulation, and in some species multiple matings are required to initiate ovulation [27]. It is not clear whether females must rewarm before mating can occur, but even if they do it would seem unlikely that they could exert a pre-copulatory choice. If torpid females do not have any pre-copulatory choice this would provide strong selection for the female to mate again - to ‘trade up’ - if she subsequently encounters a better quality male [22], which in turn would select for mate guarding by males. It is also not clear why some females should re-enter torpor after mating. For female 0118 (Figure 2) the successful mating was not particularly early – it would have been in the middle of the normal mating season [12]. Re-entering torpor would be expected to prolong the gestation period, and for those egg-laying events which occurred less than 20 days after the final arousal from hibernation (Figure 2) the time from mating, as estimated from temperature records was 26–30 days (average 28.1, *n* = 4). As females will mate when pregnant it is possible that a female which has been mated while torpid, and thus had no pre-copulatory choice, may extend her gestation period by re-entering hibernation to increase the possibility of being found by a more desirable male, allowing her the option of abandoning the first pregnancy.

This study raises a number of questions that should be testable when we have sufficient microsatellites to establish paternity [28]. What determines successful male parentage? Do males which mate with the largest number of females dominate paternity, or do females, despite mating with several males, have preferences? It is possible that successful male parentage is related to major histocompatibility complex (MHC) compatibility. Investigation of this aspect of mate choice is dependent on the development of MHC typing for the species.

## Materials and Methods

### Ethics Statement

This work was carried out under permit from the Tasmanian Department of Primary Industries, Water & Environment, and the University of Tasmania Animal Ethics Committee, and complies with the Tasmanian and the Australian Code of Practice for the Care and Use of Animals for Scientific Purposes (2004).

### Study site and animals

Fieldwork was conducted on a 12 km^2^ site on a grazing property in the southern midlands 50 km north of Hobart, Tasmania [12]. Between 1996 and 2006 we had tagged 180 echidnas in this area with passive implantable transponder (PIT) tags (LifeChip, Destron-Fearing, St. Paul, MN, USA). At the start of 2007 six echidnas (3M, 3F) already had tracking transmitters (Bio Telemetry Tracking, South Australia) glued to the spines. Over the two years of this study we found 70 animals (26M, 31F, 13 juveniles), 37 of which had been tagged previously. Forty-eight were found while slowly driving around the property in a 4WD vehicle, 22 (5F, 17 M) were found in mating groups when we were tracking other animals. All new animals were tagged, and tracking transmitters attached to 14 females and 9 males. A small temperature logger (iButton, DS1922L, Maxim Integrated Products, Inc. Sunnyvale, California), was glued to the tracking transmitter. The camera trap was an Olympus digital camera (model) with a passive infrared motion detector (Archipelago Consulting Ltd, Westerway, Tasmania, Australia), which we were able to position above a female echidna while she was hibernating in a hollow tree, and later in a tree stump.

### Internal temperature logger data

Details of loggers and surgical procedures are described in Nicol et al. [17], and ten of the data points shown in Figure 3 were published in that study while nine data points were from loggers that were part of that study but downloaded subsequently.

### Recovery of sperm from the female reproductive tract

Urogenital smears were collected by inserting a soft flexible catheter (6 mm diameter) into the cloaca while the female was anaesthetized under light isoflurane anaesthesia. The lubricated catheter was inserted approximately 5 cm into the urogenital tract, bringing the tip into close proximity to the opening of the paired uteri. A nylon bristled DNA buccal cell collection brush (MasterAmp™ Buccal Swab Brush, Epicentre Technologies, Madison WI, USA) was then advanced through the catheter until the bristles were just beyond the end of the catheter. The brush was then withdrawn from the catheter and wiped across a microscope slide. Slides were then air-dried and stained (Rapid Diff, Australian Biostain Pty. Ltd., Victoria, Australia). The appearance of spermatozoa recovered from the female reproductive tract changed quite significantly over several days of repeated sampling. Sperm sampled immediately after mating had at least 4 curves along their length. With increasing time in the female tract the sperm had fewer and fewer curves, and after 4 to 5 days began to break into fragments.

Ultrasonography was carried out in the field, using a MyLab30CV portable ultrasound with linear probe (Esaote, Genova, Italy). For ultrasound examination echidnas were lightly anaesthetized with isoflurane-oxygen, and placed on their back.
